# A new therapeutic basis for treating Li-Fraumeni Syndrome breast tumors expressing mutated TP53

**DOI:** 10.18632/oncotarget.183

**Published:** 2010-10-17

**Authors:** Robert I. Glazer

**Affiliations:** Department of Oncology, Lombardi Comprehensive Cancer Center, Georgetown University School of Medicine, Washington, DC 20007, USA

Li-Fraumeni Syndrome (LFS) is a rare autosomal dominant disorder characterized by germline mutations in *TP53* and the early onset of multiple forms of cancer, including breast cancer [1,2]. As predicted by the Knudsen two-hit hypothesis, mutation or inactivation of one allele results in somatic inactivation of the second allele, ie. loss of heterozygosity [3]; however, one-hit effects without LOH can also account for an increased growth advantage and tumorigenicity [4]. The National Comprehensive Cancer Network (NCCN) provides guideline recommendations for screening LFS family members to aid early detection of tumors. However, many LFS families remain undiagnosed as a result of a lack of atypical histopathological presentations that can be used to identify these families. Additionally, diagnosis is complicated by the fact that almost all LFS-associated TP53 mutations are missense, which often make clinicians reluctant to make a diagnosis of LFS due to the inherent ambiguity of classifying missense variants. Therefore, better molecular diagnostics for LFS family members are needed so that they can benefit from screening. Because TP53 mutations are among the most common in all tumors, LFS diagnostics have great potential to be applied to early detection of sporadic tumors in patients whose tumors acquire somatic *TP53* mutations. Herbert *et al.* now report differences in the molecular signature associated with two breast epithelial and stromal cell lines derived from LFS patients with different TP53 mutations. They assess differences between these two LFS genotypes and normal control tissue by gene array analysis, and compare the relative sensitivity of target genes to TP53-modifying drugs.

**Figure 1:**
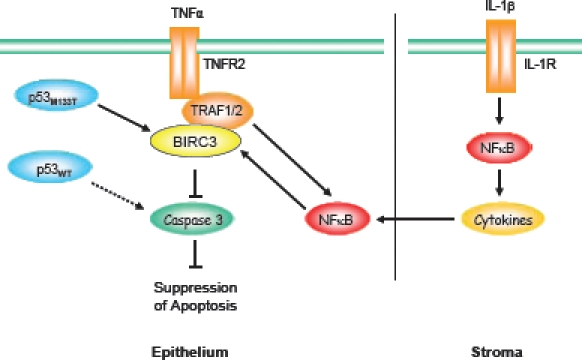
Survival pathways associated with the Li-Fraumeni mutated TP53 phenotype Li-Fraumeni Syndrome (LFS) breast epithelial cells heterozygous for *TP53* mutation M133T exhibit a marked upregulation of BIRC3 expression. BIRC3 associates with the TNFα receptor 2 (TNFR2)-associated proteins, TRAF1 and TRAF2, to inhibit caspase-3 activation and block apoptosis. Additionally, TRAF1/2 upregulates expression of transcription factor NFκB, which in turn increases BIRC3 expression. LFS stromal cells exhibit upregulation of IL-1β, which induces NFκB, and the secretion of cytokines that further perpetuate NFκB expression and pro-survival signaling.

Herbert *et al.* demonstrate that breast epithelial cells with the *TP53* M133T mutation (LFS-50) exhibit a marked increase (20-40-fold) in expression of the antiapoptotic gene, BIRC3, as well as an increase in IL-1β gene expression in stromal cells. This contrasts with little or no changes in the expression of these genes in epithelial and stromal cells (LFS-IUSM) derived from a patient with the frameshift mutation. This remarkable difference suggests that cells heterozygous for mutated TP53, in contrast to those expressing wild-type TP53, may have a survival advantage. BIRC3 associates with the TNFR2-associated factor, TRAF2, which mediates inhibition of caspase-3 [5] (Fig. 1). TRAF2 also promotes increased NFκB expression, a transcription factor that upregulates BIRC3 expression [6]. Since stromal cells associated with the mutated TP53 phenotype exhibit elevated expression of IL-1β, which also activates NFκB signaling and the expression of inflammatory cytokines, this suggests a scenario, whereby cytokine secretion by adjacent stromal cells can exacerbate the antiapoptotic signaling pathway in epithelial cells (Fig. 1). Interestingly, the high frequency of osteosarcomas in heterozygous TP53 mice [7], as in LFS patients [2], exhibit a similar increase in Birc3 gene expression, and a dependency on this gene for tumor growth.

Herbert *et al.* also provide a basis for a therapeutic approach that may selectively inhibit tumors in LFS patients expressing the mutated TP53 phenotype. Treatment of LPS-50 cells with both CP-31398 and PRIMA-1, drugs believed to interrupt signaling by mutated TP53 and convert the mutated TP53 conformation to the wild-type conformation [8,9], produced a synergistic inhibitory effect on BIRC3 expression, and a reduction in cell growth. These results imply that therapy targeting mutated TP53 may selectively induce apoptosis in tumors from patients with this genotype. These studies also suggest that patients with the inflammatory gene signature in stromal tissue may derive additional benefit from treatment with anti-inflammatory therapy to interrupt the feed forward pro-survival cycle induced by mutated TP53.
